# Detecting trap states in planar PbS colloidal quantum dot solar cells

**DOI:** 10.1038/srep37106

**Published:** 2016-11-15

**Authors:** Zhiwen Jin, Aiji Wang, Qing Zhou, Yinshu Wang, Jizheng Wang

**Affiliations:** 1 Beijing National Laboratory for Molecular Sciences Key Laboratory of Organic Solids Institute of Chemistry Chinese Academy of Sciences, Beijing 100190, P.R. China; 2 Department of Physics, Beijing Normal University, Beijing 100875, China

## Abstract

The recently developed planar architecture (ITO/ZnO/PbS-TBAI/PbS-EDT/Au) has greatly improved the power conversion efficiency of colloidal quantum dot photovoltaics (QDPVs). However, the performance is still far below the theoretical expectations and trap states in the PbS-TBAI film are believed to be the major origin, characterization and understanding of the traps are highly demanded to develop strategies for continued performance improvement. Here employing impedance spectroscopy we detect trap states in the planar PbS QDPVs. We determined a trap state of about 0.34 eV below the conduction band with a density of around 3.2 × 10^16^ cm^−3^ eV^−1^. Temperature dependent open-circuit voltage analysis, temperature dependent diode property analysis and temperature dependent build-in potential analysis consistently denotes an below-bandgap activation energy of about 1.17–1.20 eV.

PbS colloidal quantum dots (PbS QDs) are attractive materials for next generation photovoltaic devices due to their facile solution processing, low material cost, long term air stability and possibility of tailoring their optoelectronic properties by tuning size, composition and surface chemistry^1^,^2^,^3^,^4^. However, PbS QDs in solution are typically surrounded by long aliphatic ligands and once they form solid film, the long ligands act as barriers for charge transfer and transport between neighboring QDs^5^,^6^,^7^. The ligand-exchange procedure, which is used to remove such long ligands can create many surface traps such as vacancies and dangling bonds^8^,^9^, these traps assist carrier recombination, and hence seriously limit the device performance^10^,^11^. Great efforts have been put in developing surface passivation approaches to reduce the traps, and power conversion efficiency (PCE) of PbS quantum dot photovoltaics (QDPVs) has been significantly improved (over 10%)^12^,^13^,^14^,^15^. Nevertheless, the achieved PCE is still far below the expected and the surface traps remains a key limiting factor for PbS QDPVs^16^,^17^,^18^.

Trap states in the PbS QDPVs has been carefully investigated to understand how they limit the device performance. For examples, Ip, A. H. *et al*. quantified the density of mid-gap trap states in PbS QDs solids and show that the performance of PbS QDPV is limited by electron-hole recombination via these states^19^. Bozyigit *et al*. studied trap states in meta-semiconductor-metal diodes (ITO/PbS-EDT/LiF/Al), and concluded that the trap states is independent of the ITO/PbS QDs interface and can even pin the Fermi-level^20^,^21^,^22^,^23^. Recently Chuang *et al*. demonstrated the presence of trap states filling in their recently designed planar PbS QDPVs (ITO/ZnO/PbS-TBAI/PbS-EDT/Au) by using photoluminescence and electroluminescence spectroscopy measurements (TBAI: Tetrabutylammonium iodide, EDT: 1,2-ethanedithiol), and concluded that these trap states are most likely the origin of high open circuit voltage deficit^24^. Despite the intensive studies, origins and roles of trap states in PbS QDPVs are still not well-understood.

Here we employ Impedance Spectroscopy (IS) to obtain the information of the trap states in our fabricated device with currently most advanced architecture of ITO/ZnO/PbS-TBAI/PbS-EDT/Au (shown in Fig. 1(a)) 12,13. In such a device the diode property is from ZnO/PbS-TBAI heterojunction, and the PbS-EDT layer acts as an electron-blocking layer between the PbS-TBAI layer and the Au anode^24^. For the ZnO/PbS-TBAI heterojunction, after suitable illumination (mainly for the UV region), the ZnO doping density is much higher than the PbS-TBAI doping density, thereby can be regarded as a N^+^ P abrupt junction, with the depletion region locates in the PbS-TBAI film^25^,^26^. In our study we use AM 1.5 illumination (10 min AM 1.5 illumination) to generate a N^+^ P ZnO/PbS-TBAI heterojunction, and then performed various measurements including current-voltage and capacitance-voltage under various temperatures to gain information in the PbS-TBAI film. Without the pre-illumination various expected parameters cannot be extracted and various curves were actually seriously distorted (shown in Supporting Information, Figs S1–S4), similar with what Willis *et al*. described^25^. IS were tested under a variety of temperatures between 193 K and 293 K under short-circuit condition.

## Results and Discussion

**Figure 1:**
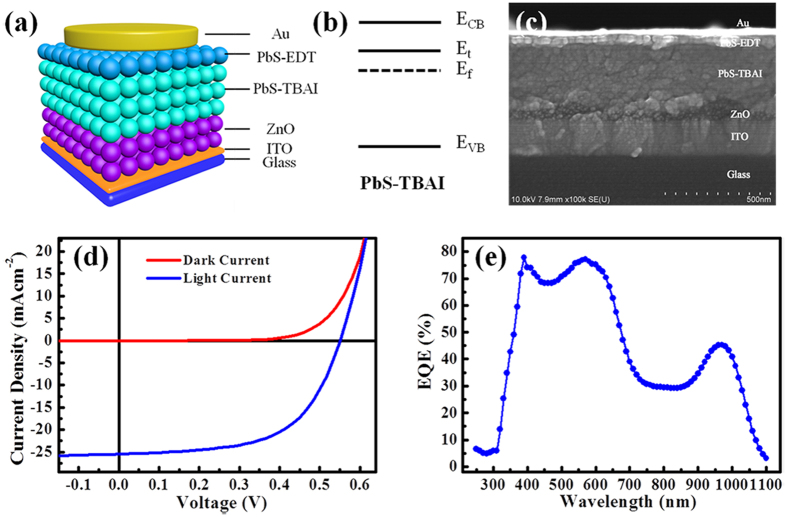
(**a**) The device architecture. (**b**) Schematic of the states in the band gap of the PbS-TBAI semiconductor, with the conduction band (E_CB_), valence band (E_VB_), the trap state (E_t_) and Fermi level (E_f_). (**c**) The cross-sectional SEM image. (**d**) J-V characteristics. (**e**) EQE spectra.

**Figure 2:**
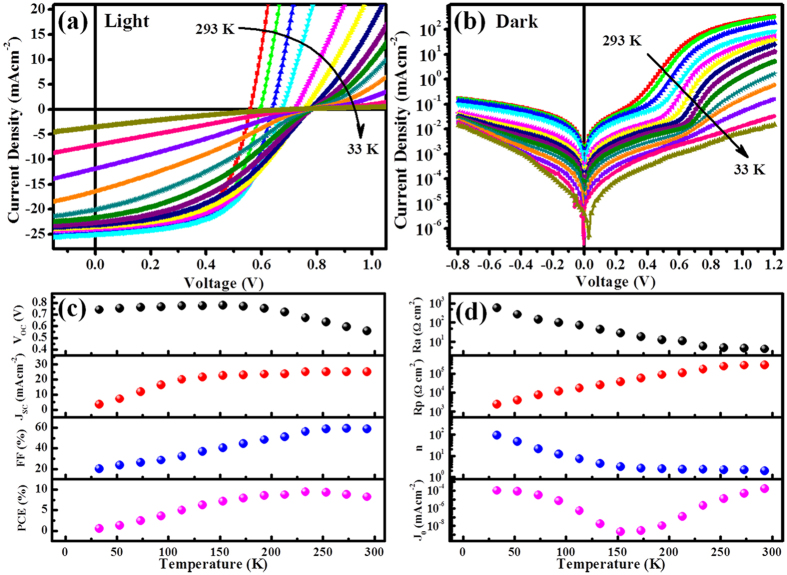
(**a**) Temperature-dependent J-V characteristics under simulated AM 1.5 illumination. (**b**) Temperature-dependent J-V characteristics in dark. (**c**) *V*_OC_, *J*_SC_, *FF* and *PCE* versus temperature. (**d**) *R*_a_, *R*_p_, *n* and *J*_*0*_ versus temperature.

Figure 1(b) shows the schematic of the states in the band gap of the PbS-TBAI semiconductor (E_CB_: the conduction band, E_VB_: valence band, E_t_: trap state, E_f_: Fermi level). Figure 1(c) is the cross-sectional scanning electron microscopy (SEM) image of the device. The current density-voltage (J-V) curve under AM 1.5 illumination at room temperature is given in Fig. 1(d). The short-circuit current density (J_SC_), open-circuit voltage (*V*_OC_) and fill factor (*FF*) are 25.03 mAcm^−2^, 0.559 V, and 58.67%, respectively (seen in Table 1). The *PCE* of 8.21% is comparable to the reported device^12^,^24^. The external quantum efficiency (*EQE*) spectrum of the device is displayed in Fig. 1(e). The light and dark J-V curves of the device under various temperatures (33~293 K with a step of 20 K) are shown in Fig. 2(a,b), respectively. From the dark J-V curves, two parameters characterizing the property of diodes, namely ideality factor (*n*) and saturation dark current density (*J*_*0*_), can be extracted with Shockley diode equation (1) listed below^23^,^27^:


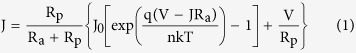


where *R*_p_ is the parallel resistance, *R*_a_ is the series resistance, q is the elementary charge, *V* is the output voltage, k is the Boltzmann constant, *T* is the temperature. In order to facilitate comparison, all the parameters for the devices extracted from J-V measurements under different temperatures are listed in Table 1 and plotted in Fig. 2(c,d). Figure 2(c), it is seen that *V*_OC_ increases with decreasing temperature till 153 K, and then begins to saturate and later drops a little. The trend has also been observed by other groups, and can be attributed to low carrier mobility in QD layers under low temperatures^28^,^29^. In Fig. 2(c,d), it is shown that *J*_SC_, *FF* and *R*_p_ decrease with decreasing *T* and *R*_a_ increases with decreasing *T*. These all can be attributed to the limited carrier mobility under low temperatures, which hampers carrier extraction and enhances recombination. The *PCE* increases from 8.21% to a high value 9.41% when temperature decreases from room temperature to 233 K, and then begins to drop. It is worth noting here that n and *J*_*0*_ show interesting trend with *T*. Generally, the values of *n* for the device increase exponentially when *T* is lower than 193 K. *J*_*0*_, meanwhile, decreases with decreasing *T* from 293 K to 193 K, and then starts to increase. From the reverse bias region of the dark current curves in Fig. 2(b), it can be seen that the reverse dark current decreases with decreasing *T* in the whole temperature range of 33~293 K, which should be the reasonable trend. The very large *n* and the enhanced *J*_*0*_ in the temperature range of 33 to 193 K could be caused by the *R*_a_. Because at low temperature, *R*_a_ becomes larger and larger, which is comparable to *R*_p_, and hence the Shockley diode equation cannot be simplified into the form of equation (1) and more voltage drops on *R*_a_. The high-quality Figures for Fig. 2(c,d) are also given in Supporting Information (Fig. S5). Therefore, the parameter extraction is meaningless when *T* < 193 K. So we focus our attention on carrier recombination mechanisms and discussed in the range of 193 K to 293 K.

**Figure 3:**
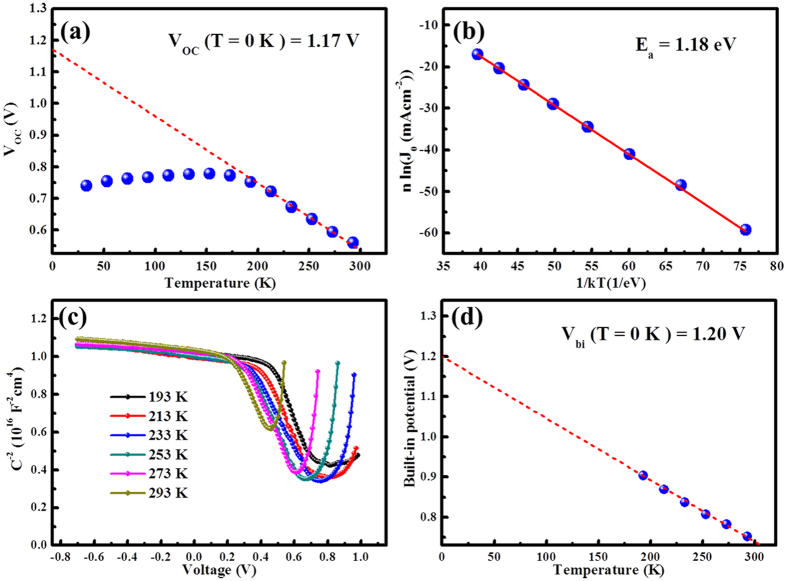
(**a**) Temperature dependent *V*_OC_. (**b**) *n* ln(*J*_*0*_) versus 1/k*T*. (**c**) C^−2^-V result (Mott-Schottky plot) under different temperatures. (**d**) Built-in potentials extracted from by C^−2^-V plot.

Figure 3(a) displays the measured *V*_OC_ versus *T*, by extrapolating the *V*_OC_ to 0 K, we obtain the maximum possible quasi-Fermi level separation achievable where screening effect of thermally generated electrons/holes is fully avoided: *qV*_OC_ = 1.17 eV (*qV*_OC_ is equal to the electron and hole quasi-Fermi level separation^26^,^30^). As we know *V*_OC_ can be written as^24^:


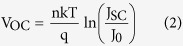


and for a single thermally activated carrier generation-recombination mechanism, *J*_*0*_ can be expressed as equation (3):


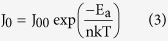


where *J*_*00*_ is the prefactor, *E*_a_ is the activation energy. By substituting equation (3) into (2), *V*_OC_ can be immediately expressed as straightforward form:


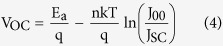


From which *E*_a_ can be directly determined as *qV*_OC_ at *T* = 0. Meanwhile equation (3) can be simply reorganized into equation (5):


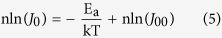


and hence from the dark J-V curves at different temperatures, *E*_a_ can be extracted, which is 1.18 eV here (shown in Fig. 3(b)), in good agreement with the value (1.17 eV) extracted from the *V*_OC_-*T* plot. This consistence confirms that the ZnO/PbS-TBAI junction dominates the *V*_OC_. It is well-known that an activation energy equal to the absorber bandgap implies the dominance of bulk generation recombination in the absorber. An activation energy smaller than the bandgap often implies the significance of trap-assisted recombination^31^. Here *E*_a_ (1.18 eV) is smaller than the optical bandgap of 1.34 eV (determined from the first exciton absorption peak of 927 nm), this suggests that *V*_OC_ is affected by recombination either from ZnO/PbS-TBAI interfacial states or trap state in PbS-TBAI QDs layer.

Figure 3(c) shows the Capacitance-Voltage (C^−2^-V) plots of the device in the temperature range of 193 K to 293 K (The capacitance cannot respond promptly with the AC signals when T < 193 K, which could also be induced by the limited carrier mobility at low temperatures). From the C^−2^-V plot, the built-in potential can be extracted based on the Mott-Schottky equation^25^:


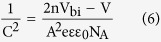


where *V*_bi_ is the built-in potential, *V* is the applied voltage, *A* is the device area, *N*_A_ is the doping concentration, *ε* is the relative permittivity, and *ε*_0_ is the permittivity of vacuum. It can be seen that *V*_bi_ increases linearly with decreasing temperature (Fig. 3(d)). The built-in potential at 0 K can be obtained from the extrapolated intersections of the fitting line with the y axis. As can be seen, the built-in potential at 0 K is 1.20 V which is in quite good agreement with extracted *V*_OC_ at 0 K from the *V*_OC_-*T* plot and *E*_a_/*q* obtained from equation (5).

**Figure 4:**
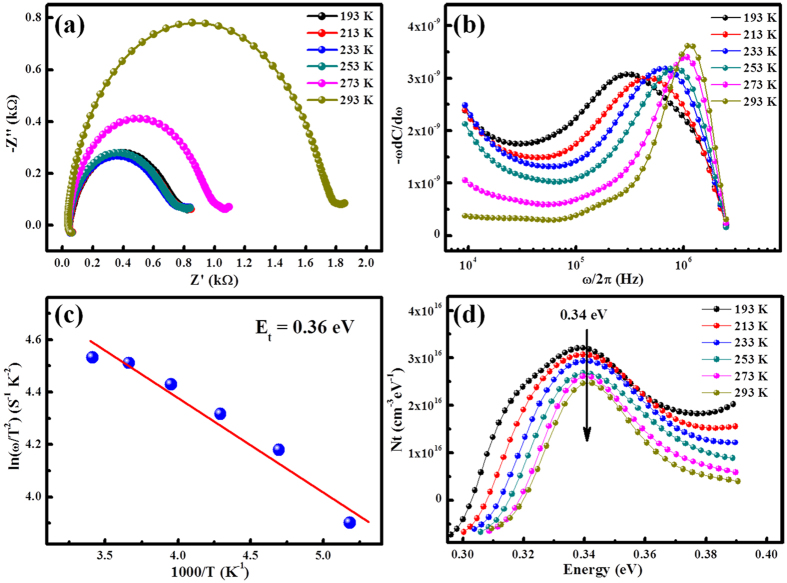
(**a**) Temperature dependent IS (with a step of 20 K) under 0 V bias at simulated AM1.5 illumination. (**b**) −*ω*d*C*/d*ω* versus *ω*/2π. (**c**) Arrhenius plot for the estimation of the detect activation energies. (**d**) Densities of trap states under different temperatures.

Impedance Spectroscopy (IS) was used to analyze the dynamics of charge transfer and recombination in the device. Figure 4(a) shows the cole-cole plot (Z′- Z: impedance imaginary part-impedance real part) with temperature varying from 193 K to 293 K with a step of 20 K. All the curves intersect with lateral axis at about 60 Ω at high frequencies region, which represents for series resistance of the devices^32^. While at low frequencies region, these plots intersect with lateral axis at different points which is related to the recombination resistance. The larger the recombination resistance, the less carrier recombination inside the device^32^. It is seen that the recombination resistance (the diameter of the Z′-Z semi-circle) decreases rapidly from 1.8 kΩ to 0.8 kΩ as the device temperature decreases from 293 K to 193 K. As told above, the trend has also been observed by other groups, and can be attributed to low carrier mobility in QD layers under low temperatures^28^,^29^. We believe this is induced by the reduced mobility with decreasing temperature, which would slow down carrier transport and enhance carrier recombination inside the device.

The defect distribution at an energy *E*_*ω*_ (below the conduction band and equal to *E*_*t*_) can be expressed by equation (7)^33^:


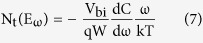


*V*_bi_ is the built-in potential (which has been obtained by C-V measurements above), *W* is depletion width, *ω* is angular frequency. The method consists of calculating the derivative of the frequency dependence of the capacitance and scaling the frequency into an energy axis. *C(ω)* can be written as^34^:


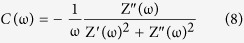


from which *C(ω)* data can be obtained from the measured impedance data *Z(ω)*. Thus, the differential capacitance *ω*d*C*/d*ω* versus *ω* can be calculated and the result is depicted in Fig. 4(b). It is seen that the differential capacitance has a peak for each temperature, which corresponds to the characteristic frequency *ω*_*0*_. Moreover, the peak shifts to low angular frequency as the temperature decreases. The relationship between the angular frequency ω and the energy *E*_*ω*_ can be described by equation (9) and equation (10)^35^,^36^:


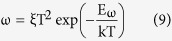



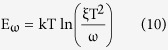


where *ξ* corresponds to the emission prefactor. According the equation (10), the active energy of trap states (*E*_*ω*_ = *E*_*t*_) was extracted from an Arrhenius plot of maxima ln(*ω*_*0*_/*T*^2^) vs 1000/*T* as shown in Fig. 4(c). The value of *E*_*t*_ is 0.36 eV which is similar to the reported value^20^,^23^. A attempt frequency *ν*_*0*_ = 2.88 × 10^12^ s^−1^ (300 K) is also obtained from which capture cross section and capture radius can be calculated to be *σ* = 1.03 × 10^−13^ cm^2^ and *r* = 1.81 nm, respectively.

The depletion width can be obtained by equation (11):


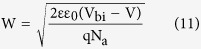


Therefore, the defect distribution can be determined by calculating the derivative in a *C(ω)* measurement and the results was depicted in Fig. 4(d). The density of defect states has a Gaussian distribution, and the width of these Gaussian distributions is on the order of thermal energy (about 26 to 35 meV). The peak with activation energy at about 0.34 eV is quite consistent with the value of *E*_*t*_. The density of defects at the peak is around 3.2 × 10^16^ cm^−3^ eV^−1^ at 193 K (the overall trap density is 1.12 × 10^16^ cm^−3^), and as the temperature increases, the density is reduced. The value and the phenomenon are in agreement with the previously reported^37^,^38^.

The extracted trap state energy level of 0.34 eV and trap state density of 3.2 × 10^16^ cm^−3^ eV^−1^ are consistent with what Bozyigit *et al*. reported^20^. Chuang *et al*. determined a trap state of 0.26 eV below the conduction band^24^. In another paper, Bozyigit *et al*. systematically investigated carrier transport and recombination as a function of QD size in PbS-EDT QD solar cells^23^. They have found that the energy depth of the traps is a function of QD bandgap, the larger the bandgap, the deeper (the larger E_t_) the trap state. In their study, (0.2 to 0.5 eV, E_t_ has been observed when bandgap is tuned from 1.22 to 1.85 eV). And the trap state density is logarithmically dependent on the bandgap. Theoretical calculations have shown that a stoichiometry with slight excess of lead favors a donor-type defect at around 0.4 eV below the conduction band^39^. Although ref. 39 does not calculate the size dependence of the trap-state energy, ref. 40 shows an increase of the trap energy with larger bandgap (smaller size)^39^,^40^. Our dot has a size around 3.2 nm, based on the experimental formula^41^:


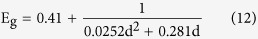


A bandgap of 1.27 eV can be obtained, which is lower than the optical bandgap obtained by the first exciton absorption peak. The QD PbS layer has a distance of about 0.3 nm (the length of TBAI), so trap number per QD is about 1.05 × 10^−3^. Based on the reports that trap number lies in a wide range of 10^−2^ to 10^−4^ per dot^42^,^43^, the very low trap density per dot in our device could be induced by the illumination where a portion of trap states could be filled by the photo carriers. It is worth to note here that although our dot size and bandgap is similar to what Chuang *et al*. reported, the trap state energy is different (0.34 vs 0.26 eV), and although our trap state energy and density is similar with what Bozyigit *et al*. reported^20^, the capture cross section and hence the capture radius are much larger. The consistencies and inconsistences of our results with the reported hint that origins of trap states in PbS QDs are very complicated and continued studies are required to deeply understand them.

In summary, we investigated trap states in short-circuited planar PbS QDPVs with the planar architecture of ITO/ZnO/PbS-TBAI/PbS-EDT/Au. A defect trap state of about 0.34 eV with a density of 3.2 × 10^16^ cm^−3^ eV^−1^ is determined. Temperature dependent J-V and C-V measurements consistently indicate that trap states greatly influence the diode ideality factor n and saturation dark current density J_0_ and build-in voltage *V*_bi_, which translates into large open-circuit voltage loss, photocurrent reduction and fill factor cut. Hence significantly reducing/illuminating trap states in the QD layer is the key to largely improving the device performance of QDPVs.

## Experimental

### Material Preparation

PbS and ZnO colloidal quantum dots were synthesized using previously reported methods^44^,^45^. The concentrations of the PbS dispersed in octane and the ZnO dispersed in chloroform were both 50 mg/ml. The TBAI solution (10 mg/ml in methanol) and EDT solution (0.02 vol% in acetonitrile) were stirred rigorously for ca. 24 h at room temperature before use.

### Device Fabrication

The ZnO layer was spin-coated on the cleaned ITO substrate, PbS QD layers were then spin-coated (at 2500 rpm for 15 s) by layer-by-layer process on the ZnO film. To form the PbS-TBAI layer, a TBAI solution was applied to the prepared PbS layer for 60 s, followed by two rinse-spin steps with methanol. To form the PbS-EDT layer, an EDT solution and acetonitrile were used. All the spin-coating steps were performed under ambient condition at room temperature. The films were stored in air overnight and then Au electrodes (100 nm thick) were thermally evaporated onto the films through shadow masks. Before measurement the unencapsulated devices were stored in air for another day.

### J-V Characterization and EQE

J-V characteristics of the devices were measured with a computer-controlled Keithley 2400 source meter at a speed of 0.01 V/s and Newport solar simulator with 100 mW/cm^2^ illumination. EQE spectra were collected using Oriel IQE-200™ in the atmosphere. Prior to the use of the light, the spectral response and the light intensity were calibrated using a mono-silicon detector produced by the National Renewable Energy Laboratory.

### Low-temperature Measurements

The sample was mounted onto a LN_2_-coolable sample stage inside a vacuum chamber and the closed-cycle cryostat Janis CCS-150 was allowed conducting experiments in the 33–293 K temperature range with a step of 20 K by providing high-pressure helium gas to the cold head with compressor. The temperature changed at a speed of 5 K/min. Before measurement, the device was stored at each setting temperature for 10 min.

### IS Measurements

The impedance spectroscopy (IS) measurements were performed using a Zahner Zennium electrochemical workstation. The impedance spectra were recorded by applying varied AC signal from 1 Hz to 4 MHz. All the AC oscillating amplitudes were set as low as 20 mV (rms) to maintain the linearity of the response.

### CV Measurements

The capacitance-voltage (C-V) measurements were performed using a Zahner Zennium electrochemical workstation. They were recorded at a frequency of 1 kHz for extracting *V*_bi_. The AC oscillating amplitudes were set as low as 20 mV (rms) to maintain the linearity of the response.

## Additional Information

**How to cite this article**: Jin, Z. *et al*. Detecting trap states in planar PbS colloidal quantum dot solar cells. *Sci. Rep.* **6**, 37106; doi: 10.1038/srep37106 (2016).

**Publisher’s note:** Springer Nature remains neutral with regard to jurisdictional claims in published maps and institutional affiliations.

## Supplementary Material

Supplementary Information

## Figures and Tables

**Table 1:** Parameters of the device (extracted from Fig. 2a,b).

**Temp. (K)**	**J**_**SC**_**(mAcm**^**−2**^)	**V**_**OC**_**(V)**	**FF (%)**	**PCE (%)**	**R**_**a**_ **(Ωcm**^**2**^)	**R**_**p**_ **(Ωcm**^**2**^)	**n**	**J**_**0**_**(mAcm**^**−2**^)
33	3.60	0.741	20.23	0.54	559.58	2.46 × 10^3^	90.39	1.01 × 10^−4^
53	7.21	0.754	23.45	1.27	261.90	3.93 × 10^3^	46.20	8.45 × 10^−5^
73	11.92	0.762	26.17	2.38	146.97	7.65 × 10^3^	21.09	3.27 × 10^−5^
93	16.47	0.766	28.45	3.59	101.62	1.16 × 10^4^	11.75	6.68 × 10^−6^
113	20.09	0.773	32.18	4.99	71.61	1.67 × 10^4^	7.13	5.57 × 10^−7^
133	21.70	0.777	36.73	6.19	44.03	2.54 × 10^4^	4.25	1.82 × 10^−8^
153	22.67	0.778	40.29	7.11	28.08	3.69 × 10^4^	3.07	2.25 × 10^−9^
173	23.08	0.772	44.26	7.89	18.54	5.66 × 10^4^	2.58	2.92 × 10^−9^
193	23.47	0.751	48.14	8.49	13.01	8.63 × 10^4^	2.44	1.07 × 10^−8^
213	23.81	0.721	50.86	8.73	11.36	1.08 × 10^5^	2.34	1.19 × 10^−7^
233	24.98	0.673	55.97	9.41	5.90	1.71 × 10^5^	2.33	2.00 × 10^−6^
253	24.98	0.635	58.77	9.32	4.95	2.41 × 10^5^	2.25	1.22 × 10^−5^
273	24.99	0.594	58.98	8.76	4.73	2.71 × 10^5^	2.19	4.79 × 10^−5^
293	25.03	0.559	58.67	8.21	4.29	2.80 × 10^5^	2.08	1.17 × 10^−4^
