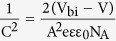# Erratum: Detecting trap states in planar PbS colloidal quantum dot solar cells

**DOI:** 10.1038/srep39725

**Published:** 2017-01-09

**Authors:** Zhiwen Jin, Aiji Wang, Qing Zhou, Yinshu Wang, Jizheng Wang

Scientific Reports 6: Article number: 3710610.1038/srep37106; published online: 11 15 2016; updated: 01 09 2017

This Article contains a typographical error in equation 6, where


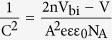


should read: