# Supplementary material for: Detecting trap states in planar PbS colloidal quantum dot solar cells
Source: Sci Rep. 2016 Nov 15;6:37106. doi: 10.1038/srep37106 (PMC5109221; doi:10.1038/srep37106)
Supplement: Supplementary Information [file srep37106-s1.pdf]

Supplementary Information

Detecting trap states in planar PbS colloidal quantum dot solar cells

Zhiwen Jin[1], Aiji Wang[2], Qing Zhou[1], Yinshu Wang[2] and Jizheng Wang[1*]

[1]Beijing National Laboratory for Molecular Sciences
Key Laboratory of Organic Solids
Institute of Chemistry
Chinese Academy of Sciences, Beijing 100190, P.R. China
E-mail: jizheng@iccas.ac.cn

[2]Department of Physics,
Beijing Normal University, Beijing 100875, China

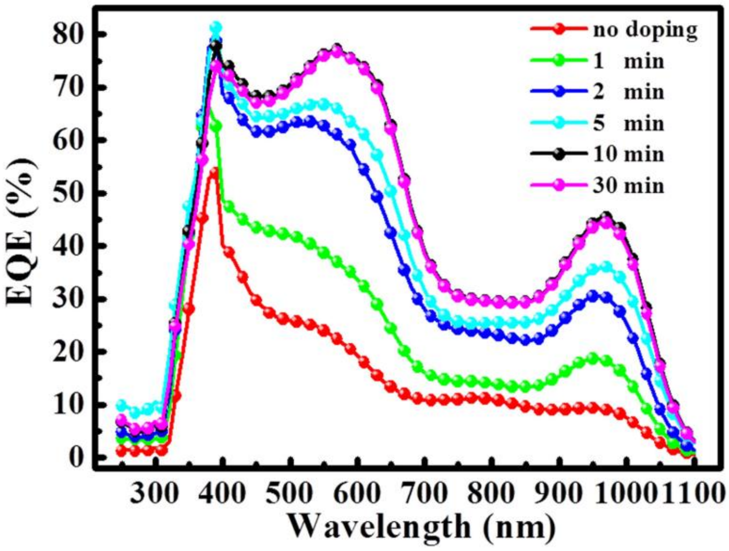

Figure S1. EQE spectra measured after different UV exposure time.

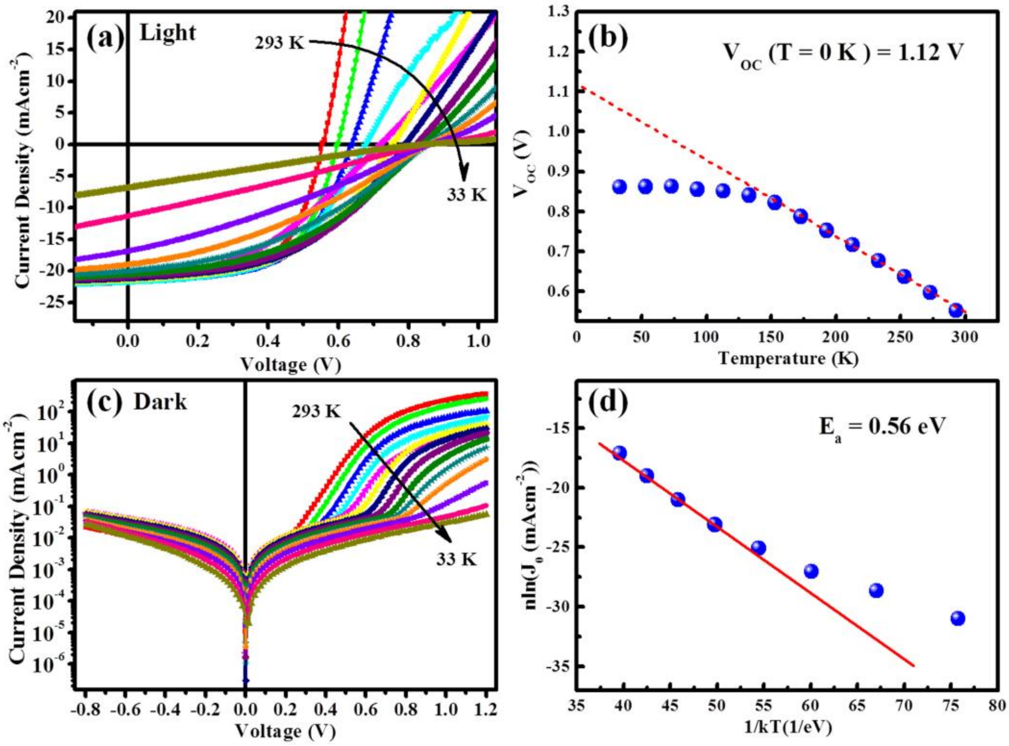

Figure S2. (a) Temperature-dependent J-V characteristics under simulated AM 1.5 illumination. (b) Temperature dependent V_{OC}. (c) Temperature-dependent J-V characteristics in dark. (d) $n\ln(J_0)$ versus 1/kT.

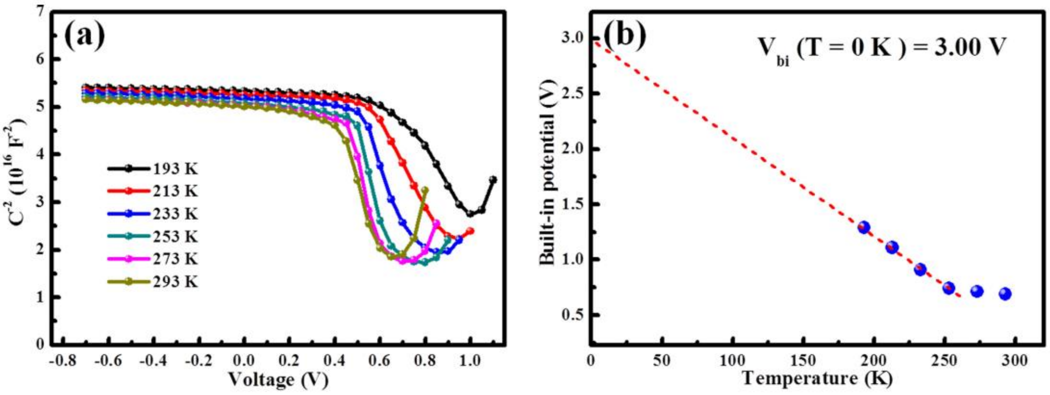

Figure S3. (a) C^{-2}-V result (Mott-Schottky plot) under different temperatures. (b) Built-in potentials extracted from by C^{-2}-V plot.

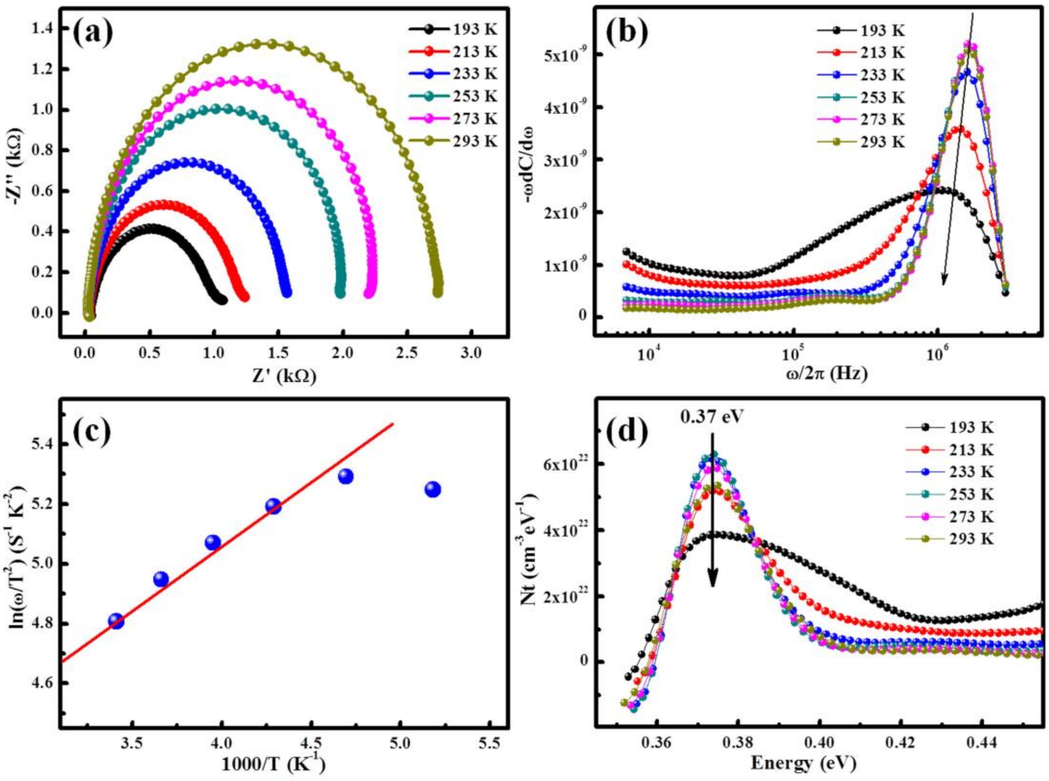

Figure S4. (a) Temperature dependent IS (with a step of 20 K) under 0 V bias at simulated AM1.5 illumination. (b) -ωdC/dω versus ω/2π. (c) Arrhenius plot for the estimation of the detect activation energies. (d) Densities of trap states under different temperatures.

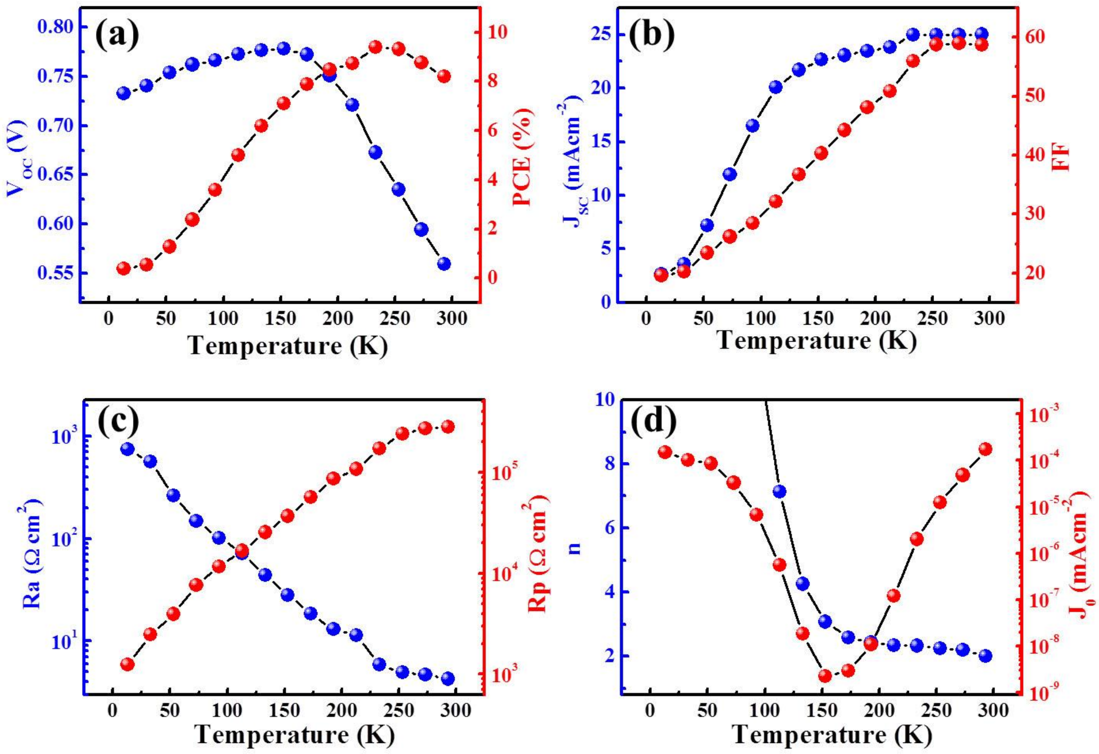

Figure S5. (a) V_{OC} and PCE versus temperature. (b) J_{SC} and FF versus temperature. (c) R_a and R_p versus temperature. (d) n and J_0 versus temperature.
